# High Rate of Elder Abuse in the Time of COVID-19—A Cross Sectional Study of Geriatric and Neurology Clinic Patients

**DOI:** 10.3390/jcm10194532

**Published:** 2021-09-30

**Authors:** Karolina Filipska, Monika Biercewicz, Adam Wiśniewski, Renata Jabłońska, Agnieszka Królikowska, Emilia Główczewska-Siedlecka, Kornelia Kędziora-Kornatowska, Robert Ślusarz

**Affiliations:** 1 Neurological and Neurosurgical Nursing Department, Faculty of Health Science, Collegium Medicum in Bydgoszcz, Nicolaus Copernicus University in Toruń, Łukasiewicza 1 Street, 85-821 Bydgoszcz, Poland; renjab_1@wp.pl (R.J.); agakrolikowska6@wp.pl (A.K.); robert_slu_cmumk@wp.pl (R.Ś.); 2 Clinic of Geriatrics, Faculty of Health Science, Collegium Medicum in Bydgoszcz, Nicolaus Copernicus University in Toruń, Skłodowskiej 9 Street, 85-094 Bydgoszcz, Poland; monika_bierc_cmumk@wp.pl (M.B.); egs@cm.umk.pl (E.G.-S.); kornelia.kornatowska@cm.umk.pl (K.K.-K.); 3 Department of Neurology, Faculty of Medicine, Collegium Medicum in Bydgoszcz, Nicolaus Copernicus University in Toruń, Skłodowskiej 9 Street, 85-094 Bydgoszcz, Poland; adam.lek@wp.pl

**Keywords:** COVID-19, elder abuse, risk factors, older adults

## Abstract

The ongoing COVID-19 pandemic is believed to have caused a sharp increase in the incidence of elder abuse (EA), including as a result of isolation, social distance combined with increased interpersonal stressors. Thus, the aim of this study is to determine the impact of the COVID-19 pandemic on the elder abuse rates and the characteristics of risk factors. A total of 347 patients hospitalized in the Department of Neurology and Department of Geriatrics at University Hospital No. 1 in Bydgoszcz were selected as subjects for the analysis. The tools used in the study are: Authors-Designed Questionnaire, the Vulnerability to Abuse Screening Scale, the Geriatric Depression Scale and the Activities of Daily Living Scale. Descriptive statistics, chi-squared tests, Spearman’s rank correlation test, and logistic regression analyses were used. In the studied population, nearly 45% of the elderly were victims of violence. This represents an increase of more than 6 percent compared to the pre-pandemic. The most common type of EA was psychological abuse (72.3%). In the final models, the risk factors include, among others, low income (OR = 3.60, 95% CI = 1.93–6.72), chronic diseases (OR = 2.06, 95% CI = 1.28–3.31), poor relationship with the family (OR = 3.26, 95% CI = 1.96–5.43), and moderate and severe depression (OR = 18.29, 95% CI = 10.24–32.69; OR = 18.49, 95% CI = 3.91–87.30, respectively). Moreover, moderate functional impairment 5.52 times more often and severe functional impairment 21.07 times more likely to predispose to EA. People who suffered from COVID-19 are 1.59 times more likely to be victims of EA (95% CI = 1.03–2.46). In this study, we saw significant increases in EA rates during the COVID-19 pandemic.

## 1. Introduction

SARS-CoV-2 is a new single-stranded RNA beta-coronavirus. It first appeared in November 2019 in Wuhan, China. In Poland, the first case was recorded on 4 March 2020. The World Health Organization (WHO) named this disease COVID-19. Moreover, on 11 March 2020, WHO announced the beginning of the COVID-19 pandemic [1]. Coronavirus disease is associated with threats to the health and life of people all over the world, not only because of the disease itself, but also its complications. This pandemic has led to a real revolution in everyday life. In order to limit its spread, it was necessary for the state to introduce isolation, the need to maintain social distance, and also to control the behaviour of citizens. Further more, change in everyday life, economic instability, loss of job, fear of illness, social distance, and complications after illness are factors that may contribute to the occurrence of neuropsychiatric disorders, including symptoms of anxiety and depression, which has been observed among the society of many countries affected by the pandemic [2,3].

Another important effect of the pandemic is the increase in the rates of violence, including in relation to the elderly [4]. Most of the older adults who become victims of violence are people who require long-term and increased care [4,5,6]. The stress theory describes caring for the elderly as a difficult and stressful activity [5]. In addition, especially during the COVID-19 pandemic, there are pressures and stresses related to work and life. All these factors contribute to an increase in the rates of violence against the elderly by caregivers. In addition, isolation itself is also a significant risk factor for abuse. Elderly or dependent people can often only interact with their perpetrators or due to quarantine, stay only with them [4,5,6].

Elder abuse (EA) (also known as mistreatment, older adult abuse or maltreatment) is defined by the WHO as “a single or repeated act or lack of appropriate action, occurring within a relationship of trust, which causes harm or distress to an older person”. According to WHO, we distinguish five types of EA: physical, sexual, psychological, and emotional abuse, financial and material abuse, abandonment, and neglect [7]. On the other hand, the most common form of EA is psychological abuse [7,8,9]. It should be noted, however, that older adult abuse is a global public health problem, and the estimated total prevalence rate is 15.7% [10]. It is believed, however, that the ongoing COVID-19 pandemic caused a sharp increase in the incidence of EA, including as a result of isolation and social distance in combination with increased interpersonal stressors [11,12]. Our research team has already conducted cross-sectional research on elder abuse from April 2017 to January 2019. It has been shown then that among 200 respondents 38.5% of older people have experienced abuse [13]. Observing the current indicators, it can be easily noticed that there has been a sharp increase in acts of of EA during the COVID-19 pandemic. Thus, there is a strong need for research on the scale and severity of the incidence of EA and emotional distress, including symptoms of depression and generalized anxiety, in different countries.

In summary, the aim of this study was to determine the elder abuse rates and identification of the most common risk factors of mistreatment in the Polish population in a hospital setting during the COVID-19 pandemic.

## 2. Materials and Methods

### 2.1. Study Design and Participants

From October 2020 to August 2021, we conducted this cross-sectional study in the Department of Neurology and Department of Geriatrics at University Hospital No. 1 in Bydgoszcz, Poland. The study included people who met the inclusion criteria: aged 65 years and older, voluntarily agreed to participate, with sufficient speech, hearing, and cognitive abilities: no dementia or Alzheimer’s disease diagnosed by a psychologist or physician. The total population of the respondents was 347.

After admission to the ward, each patient underwent psychological and neurological assessment in order to exclude cognitive impairment and dementia. The standard tool used by the psychologists in Poland is the Montreal Cognitive Assessment test and Mini-Mental State Examination and the Clock Drawing Test. All patients who met the inclusion criteria became participants in this study. As scheduled admissions were on hold for a long time during the COVID-19 pandemic, hospitals operated on an ER, and the number of hospitalized patients was very limited. Access to other hospitals was also restricted. Thus, we were able to collect only such a group of respondents.

Due to the fact that the study was conducted during the epidemic, we took special precautions. The subjects were patients of two departments: the Department of Neurology and Department of Geriatrics at University Hospital No. 1 in Bydgoszcz. Consequently, we spoke to each test person alone in a separate room. All test persons prior to admission to the hospital tested negative for SARS-CoV-2. Each of the study participants and the researchers wore masks. During the meeting, a distance of at least 2 meters was kept. In addition, all completed questionnaires were placed in a specially prepared box, where they had a grace period of about 7 days. Each of the participants completed the questionnaires independently. In the event of any questions or doubts, the researcher was at his disposal.

### 2.2. Variables and Measurements 

Before the start of the project, a pilot study was carried out on a group of 46 people in order to obtain information on the understandability of the questions included in the Authors-Designed Questionnaire (ADQ). All comments, opinions and suggested changes have been considered. Therefore, we have removed or changed some text items to the final, easy-to-understand form. The results of the pilot studies were not included in the results of this work.

The dependent variables include: elder abuse: psychological, physical, sexual and economic abuse and the risk of EA. The definitions of these variables were:

(a) Elder abuse—this research is based on the WHO definition: “a single or repeated act or lack of appropriate action, occurring within a relationship of trust, which causes harm or distress to an older person”. The study used 4 main forms of abuse:
  Psychological abuse—understood as arrogance, vulgarity; blackmail, threats; closing, isolating; insulting, criticizing; mocking; neglect [7,9];
  Physical abuse—the most visible, consisting of in inflicting physical pain, injuries, include: jerking, hitting, kicking, pushing, burning (e.g., with a cigarette) and choking [7,13,14,15];
  Sexual abuse—engaging in sexual contact without the consent or with the forced consent of the victim, provoking sexual behaviour against the will and willingness of an elderly person, e.g. rape, unwanted touch, etc. [7,14,15];
  Economic abuse—it can manifest itself on many levels, from the possibility of limiting financial independence in the distribution of one’s own retirement benefit to forcing to take a long-term loan, refusing or limiting access to shared finances, taking money away, limiting and preventing work, robbing, and destroying valuable items [7,14,15].
(b) The risk of EA—has been assessed using the most popular tool in the world, the Vulnerability to Abuse Screening Scale (VASS). It was built of 12 questions. The questions have been arranged in a closed form, and the answer options are: “yes” or “no”. It consists of 4 subscales: dependence, dejection, vulnerability, and coercion. Each subscale contains 3 items. The dependence subscale includes: item 4–6; dejection: item 7–9; vulnerability: 1–3; coercion: 10–12. There are 9 positive questions (1–3, 7–12) and 3 negative ones (4–6). The higher the score, the greater the risk of EA. The risk of abuse is considered to be a score of 3 points and more [16]. In order to conduct this study, the psychometric properties of the VASS tool were verified. The Cronbach’s alpha coefficient for the VASS scale was 0.89.

In addition to the VASS scale, the study also used: ADQ, the Geriatric Depression Scale (GDS) 15 items [17,18] and the Activities of Daily Living (ADL) Scale [19,20]. ADQ was created specifically for the purpose of this study, as no gold standard tool for assessing elder abuse has been published in Poland so far. This tool was developed on the basis of the researcher’s own experience in conducting this type of research and the available literature [9,10,13,21,22]. The reliability of the ADQ was examined by computing internal consistency coefficients. The Cronbach alpha coefficient was 0.91. Sociodemographic questions were included in the 1st part of the questionnaire and concerned: sex, age, education, marital status, family income, and place of residence. The leading question was “During the COVID-19 pandemic, have you experienced any abuse (e.g. kicking, pulling, hitting, ridiculing, pushing, insulting) in your place of residence?”. As for the various forms of EA, the respondents answered the question: “Which of the following forms of elder abuse were used against you?” selecting from the list of the abuses they have experienced. Above, in the definition of each type of violence, we have listed all the acts characteristic of a given sub-type of abuse, which were included in the ADQ. The next questions concerned, among others: the occurrence of chronic diseases, assessment of one’s health condition, feeling lonely, depressed or anxious, and having children.

### 2.3. Ethical Statement

The study was approved by the Bioethics Committee of the Nicolaus Copernicus University in Torun at Collegium Medicum of Ludwik Rydygier in Bydgoszcz, Poland (approval no. 437/2020). The study was conducted according to the Declaration of Helsinki regarding research on humans. All subjects provided informed consent to participate in the study.

### 2.4. Statistical Analysis

The statistical analysis was performed with STATISTICA version 13.1 (Dell Technologies, Round Rock, TX, USA). In the first stage, the EA rates were analysed. The chi-square test was used successively to determine the relationship between sociodemographic characteristics and the rate of older adult abuse. Finally, a logistic regression model was performed to assess the relationship between the independent variables and the incidence and the risk of EA. Statistical results with *p* < 0.05 were considered significant and the performed analyses were assessed in the 95% confidence interval (CI).

## 3. Results

The overall data of the included patients are shown in Table 1.

**Figure 1:**
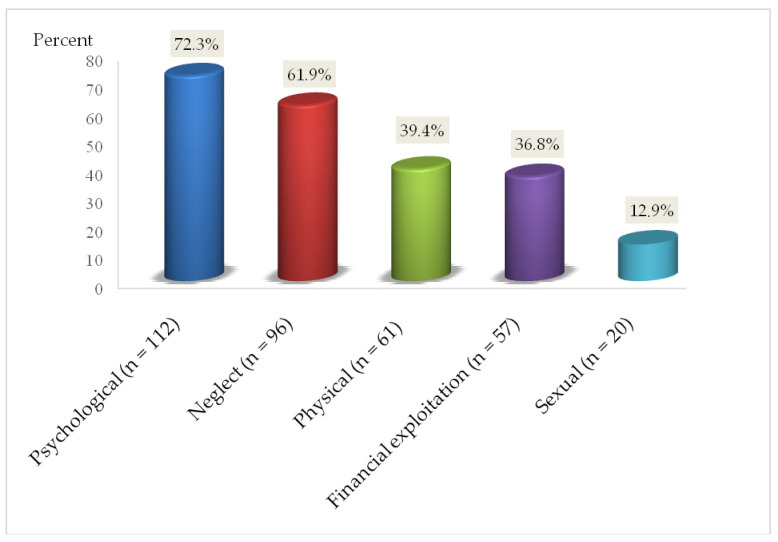
Type of elder abuse.

During the COVID-19 pandemic, nearly 45% of the elderly in the study population were victims of EA (*n* = 155). The most common type of abuse was psychological (72.3%), followed by neglect (61.9%), physical (39.4%) and economic (36.8%) (Figure 1).

The logistic regression model (Table 2) showed many variables that were important risk factors for EA. For example, women were 1.90 (95% confidence interval (CI) = 1.23–2.93) times more likely to experience acts of abuse than men. Compared to people > 70 years of age, people aged 60–65 and 66–70 were statistically more likely to be victims of EA (odds ratio (OR) = 2.35, 95% CI = 1.28–4.31; OR = 1.98, 95% CI = 1.05–3.75, respectively). It was also shown that people with higher education statistically less frequently experienced EA than people with primary education (OR = 0.32, 95% CI = 0.16–0.64). When it comes to marital status, the acts of EA were more frequent in divorced persons and widows/widowers compared to singles (OR = 4.15, 95% CI = 1.70–10.15; OR = 2.50, 95% CI = 1.20–5.25, respectively). Low income was significantly associated with an increased risk of older adult abuse (OR = 3.60, 95% CI = 1.93–6.72). Moreover, people with chronic diseases were 2.06 times more likely to experience abuse (95% CI = 1.28–3.31). Poor relationship with the family and lack of family was also significantly related to EA (OR = 3.26, 95% CI = 1.96–5.43; OR= 3.32, 95% CI = 1.68–6.56, respectively). One of the leading risk factors also turned out to be moderate and severe depression (OR = 18.29, 95% CI = 10.24–32.69; OR = 18.49, 95% CI = 3.91–87.30, respectively). The study also showed that moderate impairment (3–4 points in ADL scale) was 5.52 times more often and severe functional impairment (≤ 2 points in ADL scale) was 21.07 times more likely to predispose patients to EA. People who suffered from COVID-19 in the past were 1.59 times more likely to be victims of older adult abuse (95% CI = 1.03–2.46).

The project also assessed the risk of EA using the VASS scale. It has been shown that in the study population nearly 46% of the elderly were at risk of abuse (VASS ≥ 3 points). Most of the factors predisposing to increased susceptibility to abuse were similar to those obtained in the assessment of the presence of EA. The exception was age and place of residence, which according to the logistic regression model were not significant risk factors for abuse. Interestingly, who the respondent lives with affects the very risk of EA For example, older people living with a son/daughter or cohabitating partner were more likely to be abused than those living with their spouse (OR = 4.41, 95% CI = 2.43–8.02; OR = 3.75, 95% CI = 1.80–7.81, respectively) (Table 2).

We found moderate, positive and significant correlation between EA and the VASS scale (*R* = 0.54; *p* < 0.05). In addition, the GDS scale showed a statistically significant correlation with the VASS scale and with the occurrence of older adult abuse (*R* = 0.68 and *R* = 0.54, respectively). Subsequently, it was observed that the ADL scale correlated significantly with both EA and VASS (*R* = −0.46 and *R* = −0.58, respectively). Moreover, the self-assessment of the health condition correlates in a statistically significant negative way only with the VASS assessment (*R* = −0.19) (Table 3).

## 4. Discussion

To the best of four knowledge we are the first to highlight the association between COVID-19 and EA’s occurrence in Poland in a hospital setting. In our study we confirmed the increase in the experience of abuse by the elderly during the COVID-19 pandemic. We emphasized that women, people aged 60–65, low socioeconomic status, chronic diseases, poor relationship with the family and lack of family, moderate and severe depression, ADL ≤ 3 and COVID-19 were factors that predispose mainly to EA and to increased susceptibility to abuse assessed using the VASS scale. Our reports additionally coincide with the evolving evidence of a surge in EA during a pandemic. Thus healthcare professionals must prepare themselves as best as possible to deal with this growing problem among their patients. We enrolled only hospitalized people. Therefore, the results of these studies cannot be strictly generalized to the entire Polish population. Further research is needed in the various settings of older adults. Our research during the COVID-19 pandemic showed that nearly 45% of the hospitalized elderly were victims of EA. On the other hand, in a cross-sectional study conducted by our team in the period before COVID-19 on a group of 200 older adults with similar inclusion criteria, it was shown that 38.5% of respondents had experienced abuse [13]. This means an increase of over six percentage points. Both the present and past findings indicate that psychological abuse is the most common form of EA [9,13]. On the other hand, Chang et al. [4] noted the occurrence of EA during the COVID-19 pandemic among 21.3% of respondents, an 83.6% increase compared to prevalence estimates prior to the pandemic. In addition, in China, a study by Du and Chen [23] found that 15.4% of the older adults were victims of EA. The conducted preliminary analyses of factors indicate an actual large increase in the percentage of victims of older adult abuse [24,25]. So far, however, only a limited number of studies have been published on the occurrence of EA during COVID-19. Therefore, our results could provide relevant and missing information in this area of research in a pandemic.

Before the pandemic, in the ABUEL study, conducted among seven European countries (Germany, Italy, Lithuania, Sweden, Portugal, Spain and Greece) among 4467 respondents aged 60–84 years old, the incidents of elder abuse and neglect was also assessed. It was shown that within 12 months, psychological abuse was experienced by 19.4% of respondents, financial exploitation—3.8%, physical—2.7%, and sexual—0.7% [26]. Interestingly, research conducted in Ireland found that the country has the lowest prevalence of EA—2.2% [27]. In turn, the highest prevalence is found in Croatia—61.1% [28]. These results prove, that the prevalence rate of elder abuse varies widely. From the few studies conducted in Poland, it can be concluded that the EA rates in Poland also remains at a high level. Research conducted by a team of psychologists from the Institute of Psychology of the Polish Academy of Sciences in Poland shows that 59.7% of respondents reported the use of at least one form of EA outside their own family, and 30.1% in their own family [29]. In turn, the study by Kołodziejczak et al. [30] found that abuse affected 40.1% of older respondents living in rural areas. Our results are consistent with those presented by other authors from many different countries. For example, in a study by Hosseinkhan et al. [31] among 683 older adults it was found that 38.5% of the respondents were victims of EA. Subsequently, Anand [32] showed that out of 1435 respondents, 35% had experienced abuse. Torres-Castro et al. [33] reported a violence rate of 35.7%, and the study group was 487. If before the pandemic the EA rates in some countries were high and now increase even more, we will be faced with a serious social problem.

Interestingly, there are some common risk factors for both fraud susceptibility and COVID-19. Certainly, these factors include comorbidities that predispose to EA [34,35] and are associated with a higher mortality rate due to COVID-19 [36]. Following this trail, it can be safely stated that disability is also a significant risk factor for EA [37] and COVID-19 [38]. Moreover, COVID-19 itself predisposes to an increase in abuse among the elderly [4,24,25]. The remaining risk factors for EA during the pandemic do not differ from those that existed before the pandemic. And these include: female gender, younger age, economic problems, city living, comorbidities, depression, disability and dependence. Our results are consistent with the results presented by other researchers [7,35,39,40,41]. Our research also indicates that statistically single people were more likely to experience abuse. In addition in the research conducted by Liu et al. [25], victims of older adult abuse reported a feeling of loneliness. Further more, a poor relationship with the family predisposes you to EA in a statistically significant way. Fraga Dominguez et al. [40] also showed that family relationships are a significant risk factor for abuse. 

Research shows that the COVID-19 pandemic has added fuel to the fire in terms of EA. It turned out to be extremely harmful to the older adults. Many of the EA risk factors presented have increased during the course of the pandemic. For example, the need for isolation and social distancing have contributed to feelings of loneliness and neglect. In addition, the elderly are aware of the dangers of falling ill with COVID-19, and have experienced a real threat to health (and sometimes life) as a result of infection. It can be assumed that they may therefore be particularly prone to developing depressive and anxiety symptoms. Consequently, it is also associated with an increased risk of EA, as many studies have identified depression as a risk factor for abuse [33,42,43,44,45,46]. Depressive disorders cause further deterioration of mental and physical functioning, loss of social position, autonomy, and, as a result, the disappearance of social relations. All these factors increase the occurrence of acts of EA. Moreover, experiencing abuse aggravates depression and increases anxiety [42,43,45]. Further more, the older adults are a group particularly at risk of complications after contracting COVID-19, which in consequence often leads to increased dependence on other people and disability, which is a significant risk factor for EA [4,12,46]. Another leading factor in fraud is the financial problems that have worsened during the COVID-19 pandemic. Mass dismissals from work, forced leaves and isolation resulted in a decline in social status among the society. Due to the fact that pensions of the elderly in Poland are often insufficient, they require financial assistance from their children or family. The emerging economic pressure, stress and economic problems of the families of the elderly are the main cause of EA [23].

We are fully aware of the limitations. The study was conducted in a limited geographical area, so be careful in drawing conclusions on the entire population. In addition, the subjects are hospitalized people, therefore future research should be extended to include a research group from various environments and different regions.

## 5. Conclusions

Overall, this study saw an increase in EA rates during the COVID-19 pandemic. Factors such as: female gender, younger age, economic problems, living in a city, comorbidities, disability and dependence, loneliness, poor relationship with the family and lack of family, moderate and severe depression, ADL ≤ 3, and COVID-19 in a significant manner influenced the occurrence of abuses. Due to the fact that so far little data on this subject has been published, it is necessary to conduct further detailed research. 

## Figures and Tables

**Table 1:** Descriptive characteristics.

Characteristics	*N* (%)
Sex	
Female	194 (55.9)
Male	153 (44.1)
Age	
65–70 years	162 (46.7)
71–85 years	118 (34.0)
>85 years	67 (19.3)
Education	
Primary	87 (25.1)
Secondary	100 (28.8)
Vocational	91 (26.2)
Higher	69 (19.9)
Marital Status	
Single (never married)	45 (13.0)
Married	103 (29.7)
In a partnership	39 (11.2)
Divorcee	43 (12.4)
Widow/Widower	117 (33.7)
Equivalent family income	
Low <233	101 (29.1)
Middle	164 (47.3)
High >465	82 (23.6)
Residency area	
City	223 (64.3)
Village	124 (35.7)
Chronic disease	
Yes	240 (69.2)
No	107 (30.8)
Depression (GDS scale)	
No	216 (62.2)
Moderate	119 (34.3)
Severe	12 (3.5)
Activities of Daily Living (ADL)	
Full function (5–6 )	212 (61.1)
Moderate impairment (3–4)	100 (28.8)
Severe functional impairment (≤2)	35 (10.1)
COVID-19 in the past	
Yes	147 (42.4)
No	200 (57.6)

**Table 2:** Logistic regression analyses of factors associated with elder abuse and vulnerability to abuse screening scale (VASS).

Characteristic	Elder Abuse			Vulnerability to Abuse Screening Scale (VASS)		
	*N* (%)	OR (95%CI)	*p*	*N* (%)	OR (95%CI)	*p*
Overall	155 (44.7)	----	----	159 (45.8)	----	----
Sex						
Male	55 (35.5)	1.00		57 (35.8)	1.00	
Female	100 (64.5)	1.90 (1.23–2.93)	0.003 *	102 (64.2)	1.87 (1.21–2.88)	0.004 *
Age						
60–65	81 (52.3)	2.35 (1.28–4.31)	0.005 *	78 (49.1)	1.66 (0.93–2.99)	0.089
66–70	54 (34.8)	1.98 (1.05–3.75)	0.035 *	57 (35.8)	1.67 (0.90–3.10)	0.101
>70	20 (12.9)	1.00		24 (15.1)	1.00	
Education						
Primary	47 (30.3)	1.00		47 (29.6)	1.00	
Secondary	47 (30.3)	0.75 (0.42–1.34)	0.338	47 (29.6)	0.75 (0.42–1.34)	0.338
Vocational	42 (27.1)	0.73 (0.41–1.32)	0.294	51 (32.1)	1.08 (0.60–1.96)	0.786
Higher	19 (12.3)	0.32 (0.16–0.64)	0.001 *	14 (8.7)	0.22 (0.11–0.45)	<0.001 *
Marital status						
Single	13 (8.4)	1.00		15 (9.4)	1.00	
Married	43 (27.7)	1.76 (0.83–3.75)	0.140	30 (18.9)	0.82 (0.39–1.74)	0.609
In a partnership	13 (8.4)	1.23 (0.49–3.11)	0.660	21 (13.2)	2.33 (0.96–5.64)	0.601
Divorcee	27 (17.4)	4.15 (1.70–10.15)	0.002 *	25 (15.7)	2.78 (1.17–6.61)	0.021 *
Widower/Widow	59 (38.1)	2.50 (1.20–5.25)	0.015 *	68 (42.8)	2.78 (1.35–5.70)	0.005 *
Equivalent family income						
Low <233	59 (38.1)	3.60 (1.93–6.72)	<0.001 *	63 (39.6)	6.34 (3.25–12.37)	0.000 *
Middle	73 (47.1)	2.06 (1.16–3.65)	0.013 *	79 (49.7)	3.55 (1.92–6.58)	<0.001 *
High >465	23 (14.8)	1.00		17 (10.7)	1.00	
Place of residence						
City	110 (71.0)	1.71 (1.09–2.68)	0.020 *	105 (66.0)	1.15 (0.74–1.79)	0.526
Village	45 (29.0)	1.00		54 (34.0)	1.00	
Chronic disease						
Yes	120 (77.4)	2.06 (1.28–3.31)	0.003 *	126 (79.2)	2.48 (1.53–4.01)	<0.001 *
No	35 (22.6)	1.00		33 (20.8)	1.00	
Loneliness						
Never or rarely	55 (35.5)	1.00		49 (30.8)	1.00	
Often	74 (47.7)	2.31 (1.45–3.68)	<0.001 *	80 (50.3)	3.27 (2.03–5.25)	<0.001 *
Very often or almost always	26 (16.8)	2.89 (1.46–5.72)	0.002 *	30 (18.9)	5.07 (2.48–10.39)	<0.001 *
Participation in family decisions						
Never or rarely	103 (66.5)	1.00		107 (67.3)	1.00	
Often	33 (21.2)	0.46 (0.28–0.78)	0.003 *	34 (21.4)	0.45 (0.27–0.75)	0.002 *
Very often or almost always	19 (12.3)	0.31 (0.17–0.57)	<0.001 *	18 (11.3)	0.26 (0.14–0.49)	<0.001 *
Relationship with the family						
Good	37 (23.9)	1.00		38 (23.9)	1.00	
Fair	9 (5.8)	0.94 (0.40–2.23)	0.890	5 (3.1)	0.43 (0.15–1.20)	0.106
Poor	80 (51.6)	3.26 (1.96–5.43)	<0.001 *	86 (54.1)	3.76 (2.25–6.27)	0.000 *
Lack of family	29 (18.7)	3.32 (1.68–6.56)	<0.001 *	30 (18.9)	3.47 (1.76–6.87)	<0.001 *
Live with						
Spouse	38 (24.5)	1.00		29 (18.2)	1.00	
Cohabitant	21 (13.5)	1.34 (0.66–2.74)	0.408	28 (17.6)	3.75 (1.80–7.81)	<0.001 *
Son/daughter	51 (32.9)	1.71 (0.97–3.00)	0.064	64 (40.3)	4.41 (2.43–8.02)	<0.001 *
Alone	45 (29.1)	1.24 (0.71–2.18)	0.444	38 (23.9)	1.41 (0.78–2.54)	0.252
Depression (GDS scale)						
No	46 (29.7)	1.00		54 (34.0)	1.00	
Moderate	99 (63.9)	18.29 (10.24–32.69)	<0.001 *	95 (59.7)	11.86 (6.90–20.45)	<0.001 *
Severe	10 (6.4)	18.49 (3.91–87.30)	<0.001 *	10 (6.3)	15.00 (3.19–70.61)	<0.001 *
Activities of Daily Living (ADL)						
Full function (5–6 )	57 (36.8)	1.00		49 (30.8)	1.00	
Moderate impairment (3–4)	67 (43.2)	5.52 (3.30–9.25)	<0.001 *	77 (48.4)	11.14 (6.33–19.59)	<0.001 *
Severe functional impairment (≤2)	31 (20.0)	21.07 (7.12–62.35)	<0.001 *	33 (20.8)	54.89 (12.71–236.9)	<0.001 *
COVID–19 in the past						
No	56 (36.1)	1.00		55 (34.6)	1.00	
Yes	99 (63.9)	1.59 (1.03–2.46)	0.035 *	104 (65.4)	1.81 (1.17–2.80)	0.007 *

*—significant dependencies.

**Table 3:** Spearman’s rank correlation test.

	Elder Abuse	VASS Assessment
	R *p*	R *p*
GDS	0.54 < 0.05	0.68 < 0.05
ADL	−0.46 < 0.05	−0.58 < 0.05
The self-assessment of the health condition	−0.06 > 0.05	−0.19 < 0.05
VASS assessment	0.54 < 0.05	------

## Data Availability

The data presented in this study are available on request from the corresponding author. The data are not publicly available due to respondents privacy.
